# Diagnostic Accuracy of Bronchoalveolar Lavage Fluid Galactomannan for Invasive Aspergillosis

**DOI:** 10.1155/2020/5434589

**Published:** 2020-11-30

**Authors:** Xun-Jie Cao, Ya-Ping Li, Li-Min Xie, Hong-Lang Zhang, Yu-Shan Qin, Xu-Guang Guo

**Affiliations:** ^1^Department of Clinical Laboratory Medicine, The Third Affiliated Hospital of Guangzhou Medical University, Guangzhou 510150, China; ^2^Department of Clinical Medicine, The Third Clinical School of Guangzhou Medical University, Guangzhou 511436, China; ^3^Department of Clinical Medicine, The Second Clinical School of Guangzhou Medical University, Guangzhou 511436, China; ^4^Key Laboratory for Major Obstetric Diseases of Guangdong Province, The Third Affiliated Hospital of Guangzhou Medical University, Guangzhou 510150, China; ^5^Key Laboratory of Reproduction and Genetics of Guangdong Higher Education Institutes, The Third Affiliated Hospital of Guangzhou Medical University, Guangzhou 510150, China

## Abstract

**Background:**

The pathogenesis of invasive aspergillosis (IA) is still unknown, but its progression is rapid and mortality rate remains high. Bronchoalveolar lavage fluid (BALF) galactomannan (GM) analysis has been used to diagnose IA. This study is aimed at making an accurate estimate of the whole accuracy of BALF-GM in diagnosing IA.

**Methods:**

After a systematic review of the study, a bivariate meta-analysis was used to summarize the specificity (SPE), the sensitivity (SEN), the positive likelihood ratios (PLR), and the negative likelihood ratios (NLR) of BALF-GM in diagnosing IA. The overall test performance was summarized using a layered summary receiver operating characteristic (SROC) curve. Subgroup analysis was performed to explore the heterogeneity between studies.

**Results:**

A total of 65 studies that are in line with the inclusion criteria were included. The summary estimates of BALF-GM analysis are divided into four categories. The first is the proven+probable vs. possible+no IA, with an SPE, 0.87 (95% CI, 0.85-0.98); SEN, 0.81 (95% CI, 0.76-0.84); PLR, 9.78 (5.78-16.56); and NLR, 0.20 (0.14-0.29). The AUC was 0.94. The BALF-GM test for proven+probable vs. no IA showed SPE, 0.88 (95% CI, 0.87-0.90); SEN, 0.82 (95% CI, 0.78-0.85); PLR, 6.56 (4.93-8.75); and NLR, 0.24 (0.17-0.33). The AUC was 0.93. The BALF-GM test for proven+probable+possible vs. no IA showed SPE, 0.82 (95% CI, 0.79-0.95); SEN, 0.59 (95% CI, 0.55-0.63); PLR, 3.60 (2.07-6.25); and NLR, 0.31 (0.15-0.61). The AUC was 0.86. The analyses for others showed SPE, 0.85 (95% CI, 0.83-0.87); SEN, 0.89 (95% CI, 0.86-0.91); PLR, 6.91 (4.67-10.22); and NLR, 0.18 (0.13-0.26). The AUC was 0.94.

**Conclusions:**

The findings of this BALF-GM test resulted in some impact on the diagnosis of IA. The BALF-GM assay is considered a method for diagnosing IA with high SEN and SPE. However, the patients' underlying diseases may affect the accuracy of diagnosis. When the cutoff is greater than 1, the sensitivity will be higher.

## 1. Introduction

Aspergillus species, as a saprotrophic fungus in soil and decaying vegetation, are widely found throughout the world [1]. Among them, *Aspergillus fumigatus* is the main cause of invasive aspergillosis [2], which is a severe disseminated fungal disease and causes high morbidity and mortality among severely immunocompromised people [3]. Invasive aspergillosis (IA) occurs not only in patients with long-term neutropenia and with a history of allogeneic hematopoietic cells or solid organ transplants but also in those who use high-dose corticosteroids or genetically severe immune defective patients [4]. The invasive fungal infections in particular are also considered a significant cause of morbidity and death in immunocompromised patients [5]. The culture and microscopy still remain the gold standard for diagnosing IA, but the lack of positive cultures in blood or tissues delays the diagnosis of this infection. This requires invasive procedures, but it is difficult to implement in some cases, such as in critically ill patients or those with thrombocytopenia [5, 6]. Therefore, it is necessary to improve the fatally invasive fungal infections caused by delayed diagnosis, and so rapid processing and reporting are regarded essential.

Galactomannan (GM) is a polysaccharide that exists in the Aspergillus cell wall, which proliferates during invasive infections and is subsequently detected in the serum and other bodily fluids [7]. The role of GM might assist in diagnosing IA and has become the focus of clinical research [8]. There have been many studies on the accuracy of bronchoalveolar lavage fluid GM in the diagnosis of IA. Therefore, the 2016 ESCMID-ECMM-ERS guidelines recommended serum and bronchoalveolar lavage fluid (BALF) GM as markers for diagnosing IA [9].

To date, many studies have assessed the accuracy of the BALF-GM test in diagnosing IA. In 2012, a systematic review of 30 clinical studies evaluated patients with IA using the BALF-GM test and concluded that the optimal threshold for the BALF-GM test was 1.0 when the sensitivity (SEN) is higher [10]. Therefore, a more systematic assessment on the accuracy of the BALF-GM test in diagnosing IA through a meta-analysis was conducted in our study.

## 2. Methods and Materials

### 2.1. Research Identification and Selection

Two investigators (XJ Cao and YP Li) searched the databases such as EMBASE, PubMed, the Cochrane Library, and Web of Science for interrelated articles published till November 9, 2019. The bibliography of the included studies was also screened. The results were then manually searched for a qualifying test. Studies that were in line with the following criteria were included: (1) provided data of two-by-two tables and (2) full-text publications. The studies were excluded if the following criteria were met: (1) insufficient data, such as meeting summaries, (2) studies with less than 10 patients which were excluded in order to avoid selection bias, (3) meta-analysis and systematic reviews, and (4) animal research.

### 2.2. Quality Assessment and Data Extraction

Two investigators (XJ Cao and YP Li) independently extracted the following information: population, study, diagnostic standard, sample size, and assay characteristics; methodological quality; and data for two-by-two tables and optical density index (ODI). During the evaluation process, if there was a difference between the evaluation results of the two investigators, we shall unify opinions through discussion. A modified quality assessment for diagnostic accuracy study (QUADAS) tool was used to assess the study quality [8].

### 2.3. Statistical Analysis

To analyze a summary estimate of BALF-GM, a BALF-GM test was constructed to cross-classify into two-by-two tables (proven+probable IA vs. no IA) and two-by-two tables (proven+probable, possible IA vs. no IA). Also, the two-by-two tables (proven+probable IA vs. possible+no IA) and the two-by-two tables (other which included not EORTC/MSG consensus criteria and proven vs. no or colonization and so on) were constructed. Based on the revised EORTC/MSG consensus criteria [11], the patients were divided into four groups according to their IA diagnosis. For studies that reported multiple cutoffs, the cutoff that provided the best performance was used. A binary regression method with 95% confidence interval (CI) was used as the main outcome indicator to assess the overall specificity (SPE) and sensitivity (SEN), and a layered summary receiver operating characteristic (SROC) curve was constructed [12]. What is more, the pooled SPE and SEN were also used to calculate negative likelihood ratios (NLR) and positive likelihood ratios (PLR) [12].

The statistically significant heterogeneity was assessed using *I*^2^ statistics and explored potential heterogeneity between studies through subgroup analysis. Subgroup analysis was performed for different cutoffs that are 0.5 to 1 and greater than 1. A funnel plot was constructed to visually check for any potential publication bias [13].

The analyses were performed using Stata statistical software package, version 12.0 (StataCorp LP, College Station, U.S.A.) and Meta-DiSc 1.4.

## 3. Results

### 3.1. Study Inclusion and Exclusion Criteria and Quality Assessment

**Figure 1:**
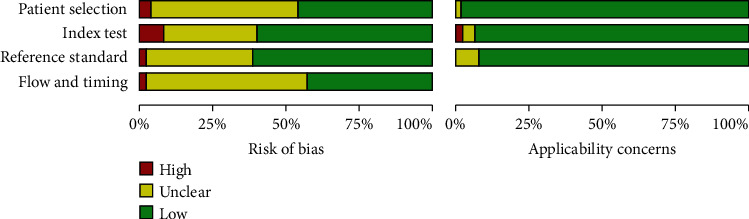
Overall quality assessment of all 65 included studies. Data are presented as stacked bars for each quality item, including modified quality assessment for studies of diagnostic accuracy (QUADAS) criteria.

Of the 896 identified studies, 65 eligible studies were eventually pooled [14–78]. The flow diagram is shown in supplementary materials (Figure S1). The characteristics of the eligible studies are presented in Table 1. Of these 65 eligible studies, 58 were cohort studies and 7 were case-control studies. The bar chart represents the quality assessment according to the improved QUADAS standard (Figure 1).

### 3.2. Analyses for Proven+Probable vs. No IA

The analyses for proven+probable vs. no IA were included in 23 studies, and 21 studies demonstrated a cutoff value of 0.5 to 1.0, and one of the two remaining had a cutoff value of 2.89 and another remained unknown. The SPE and SEN were 0.88 (95% CI, 0.87-0.90) and 0.82 (95% CI, 0.78-0.85), respectively. The NLR and PLR were 0.24 (95% CI, 0.17-0.33) and 6.56 (95% CI, 4.93-8.75), respectively. Diagnostic odds ratio (DOR) was 35.04 (23.75-51.71).

**Figure 2:**
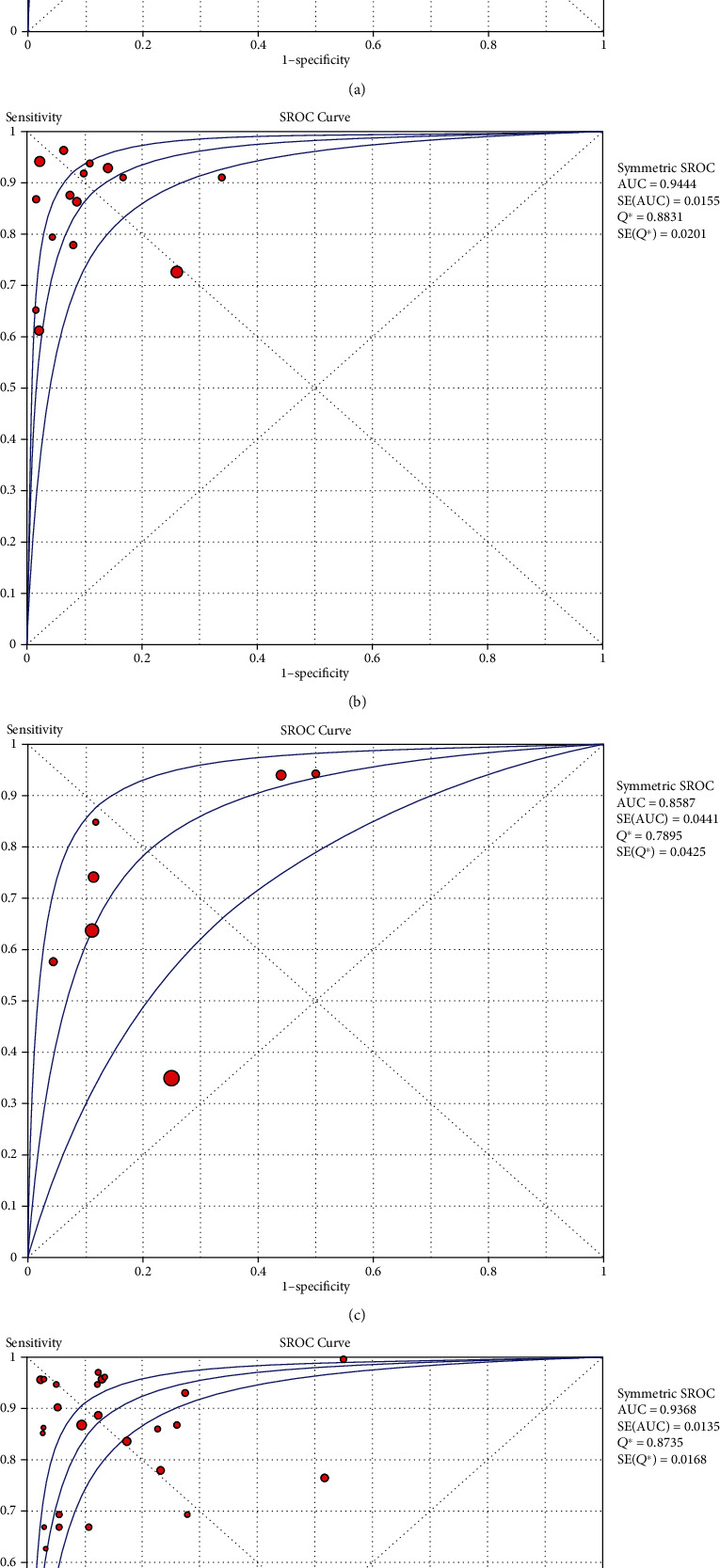
SROC curves from the bivariate model for (a) proven+probable vs. no IA, (b) proven+probable vs. possible+no IA, (c) proven+probable+possible vs. no IA, and (d) other, respectively. The smaller region (confidence contour) contains likely combinations of the mean value of sensitivity and specificity. The wider region (prediction contour) demonstrates more uncertainty as to where the likely values of sensitivity and specificity might occur for individual studies. SROC = summary receiver operating characteristic.

The SROC curve is displayed in Figure 2, representing the relationship between SPE and SEN throughout the study. The area under the SROC curve (AUC) was 0.93, which indicated that the BALF-GM assay has a high diagnostic capability.

The results of subgroup analyses for “proven or probable vs. no IA” are shown in Table 2, Figure S2, and Figure S3. The sensitivity and specificity demonstrated no significant changes. However, the heterogeneity remained significantly lower.

### 3.3. Analyses for Proven+Probable vs. Possible+No IA

The analyses of proven+probable vs. possible+no IA were included in 15 studies, in which 13 had cutoff values between 0.5 and 1.0, and the remaining two had cutoff values of 2.1 and 3, respectively. The SPE and SEN and associated 95% CIs were 0.87 (0.85-0.98) and 0.81 (0.76-0.84), respectively. The PLR and NLR and associated 95% CIs were 0.20 (0.14-0.29) and 9.78 (5.78-16.56), respectively. DOR was 72.29 (32.27-161.97). In addition to this, all measured *I*^2^ values were >50%, and this indicated significant heterogeneity among the indicators of these studies. Figure 2 displays the SROC curves, in which they represent the relationship between SPE and SEN across the studies. The area under the SROC curve was 0.94, which indicated that the BALF-GM has a high diagnostic ability.

### 3.4. Analyses for Proven+Probable+Possible vs. No IA

The analyses of proven+probable+possible vs. no IA were included in 7 studies, in which 6 of them had a threshold of 0.5 and one had a threshold of 1.0. The SPE and SEN and associated 95% CIs were 0.82 (0.79-0.95) and 0.59 (0.55-0.63), respectively. The PLR and NLR were 3.60 (95% CI, 2.07-6.25) and 0.31 (95% CI, 0.15-0.61), respectively. DOR was 14.04 (4.02-49.09).

Figure 2 shows the SROC curve, which represents the relationship between SPE and SEN throughout the study. The area under the SROC curve (AUC) was 0.86, which indicated that the resolution of BALF-GM analysis was not very high.

### 3.5. Analyses for Others

The analyses of others were included in 27 studies, in which 12 had cutoff values of 0.5 to 1, 9 had cutoff values that are greater than 1.0, one had a cutoff value of 0.4, and the remaining 4 could not be extracted. The SEN and SPE and associated 95% CIs were 0.89 (0.86-0.91) and 0.85 (0.83-0.87), respectively. The NLR and PLR were 0.18 (95% CI, 0.13-0.26) and 6.91 (95% CI, 4.67-10.22), respectively. DOR was 49.41 (27.46-88.91).

Figure 2 displays the SROC curves, and the results showed significant heterogeneity. Funnel plot results revealed no significant publication bias.

### 3.6. Publication Bias

As shown in the funnel plot, the publication bias was not significant in “proven+probable vs. no IA” and “other” groups, with *p* values of 0.43 and 0.69, respectively. The remaining studies showed significant publication bias. The results are shown in Figure S4.

## 4. Discussion

Invasive fungal infections are particularly a significant cause of morbidity and death in immunocompromised patients [2], and so the diagnosis of IA remains to be crucial. Currently, the invasive procedures mostly rely on histopathological or cytopathological evidences, which are considered the gold standard for diagnosing IA [81]. However, this diagnostic method is rarely used in certain situations, such as in critically ill patients or patients with thrombocytopenia. Due to the difficulty in diagnosing IA, a number of approaches have been developed to overcome this problem. Since 2003, there were several studies that explored the accuracy of the BALF-GM test in diagnosing IA. In 2010, Guo et al. [82] have analyzed cases with proven+probable IA vs. possible+no IA by conducting a meta-analysis, and the results achieved high accuracy of >90% for both SPE and SEN. Compared with the SEN and SPE as summarized in Guo et al.'s research, our study yielded lower SEN 0.81 (0.76-0.84) and SPE 0.87 (0.85-0.89). Four articles we included were different from Guo et al. This may be the reason for the difference. Studies showed that PLR greater than 10 and NLR less than 0.1 provided compelling diagnostic evidence, while the PLR greater than 5 and NLR less than 0.2 also provided a strong diagnostic basis to diagnose, respectively, in most of the cases [83, 84]. Although our analysis results are not so good compared with Guo et al., it still provides a strong basis for diagnosis. Similarly, the study conducted by Zou et al. showed similar results, with a PLR less than 10 but greater than 5 and an NLR of 0.15 [10]. In addition to SPE, SEN, NLR, AUC, and PLR, another test performance DOR was also reported in our study. DOR not only combines the advantages of SPE and SEN but also has superior accuracy as a single indicator [85]. The DOR was 32.27-161.97, which remained high. Based on the abovementioned results, our study also showed high accuracy for possible or no IA cases.

In the above four groups, the “proven+probable vs. no IA” group, “proven+probable vs. possible+no IA” group, “proven+probable+possible vs. no IA” group, and “others” group, the “proven or probable vs. no IA” has been implemented in many studies, which may suggest a good clinical significance. In our study, the “proven+probable vs. no IA” group showed the best SEN of 0.88 (0.87-0.90). In contrast, the “proven+probable+possible vs. no IA” group showed the lowest SPE of 0.82 (0.79-0.85), the lowest SPE of 0.82 (0.79-0.85), and the lowest AUC of 0.86. The 2019 EORTC/MSG criteria also indicated that the probable and possible categories are applicable only to immunodeficient patients [86]. In summary, this group was not so rational. Therefore, we do not recommend such grouping for patients without immunodeficiency. However, a study found that the cause of immunosuppression is not related to the EORTC/MSG classification. This study found that the classification according to the definition of EORTC/MSG criteria revealed no significant association with the cause of immunosuppression but showed a trend towards better application in stem cell transplant cases [81]. Further research needs to be done.

As shown in Table 2, in the “proven+possible vs. no IA” group, aggregated performance indicators are provided at different thresholds. However, when studies with cutoff values greater than 1 were included, the highest SEN value for BALF-GM was only 0.86. The differences in the results between the whole analysis and the subgroup analysis were mainly due to the number of studies included. When using a threshold range from 0.5 to 1.0, 15 studies were included, but when a threshold range of greater than 1 was used, only 7 studies were included. If a cutoff value of greater than 1 was used in all these studies, false-negative values might be lower or remained the same, resulting in increased or retained SEN value. Therefore, using the cutoff value of greater than 1 will have a better result.

One possible cause of heterogeneity is the use of different thresholds in different studies. The cutoff value used in this study was 0.5-1.0, and the heterogeneity was significantly reduced.

## 5. Conclusions

The BALF-GM assay is considered a method for diagnosing IA with high SEN and SPE, and if a cutoff value of greater than 1 was used, false-negative values might be lower or remained the same, resulting in increased or retained SEN value. Therefore, we recommend using the BALF-GM test to diagnose IA. Using the cutoff value of greater than 1 will have a better result.

## Figures and Tables

**Table 1:** Characteristics of 65 studies included in the meta-analysis of diagnosis of IA using BALF-GM.

Study	Diagnostic standard	Best cutoffs	Sample size	Study design	Patient population	Mean age	Male (%)
Sehgal 2019	EORTC/MSG criteria	2.5	127	Case control	Adults with MTHF	45.2	56.5
Liu 2019	EORTC/MSG criteria	0.85	190	Cohort	Adults with MTHF	NA	NA
Jenks 2019	(1) EORTC/MSG criteria; (2) a slightly modified version of the clinical algorithm described by Blot and colleagues	1	82	Cohort	Nonneutropenic adults	NA	39.0
Rozaliyani 2019	EORTC/MSG criteria	2	155	Cohort	Adults with MTHF	NA	NA
Yu 2019	EORTC/MSG criteria	2.94	184	Cohort	Nonneutropenic people	NA	0.4
Bellanger 2018	EORTC/MSG criteria	0.5	597	Cohort	Adults with MTHF	NA	NA
Imbert 2018	EORTC/MSG criteria	0.5	32	Cohort	Adults with MTHF	59.0	65.7
Hoenigl 2018	EORTC/MSG criteria	NA	28	Cohort	Adults with MTHF	60.0	28.6
Castillo 2018	EORTC/MSG criteria	0.5	106	Cohort	Adults with MTHF	55.3	65.1
Deng 2018	EORTC/MSG criteria	1.5	172	Cohort	Adults with MTHF	NA	70.2
Gupta 2017	EORTC/MSG criteria	1	71	Case control	Adults with HM	38.6	54.8
Eigl 2017	EORTC/MSG criteria	1	53	Cohort	Adults with MTHF	58.0	32.1
Taghizadeh 2017	EORTC/MSG criteria	0.5	116	Cohort	Adults with MTHF	46.0	62.9
Zhuang 2017	EORTC/MSG criteria	0.76	183	Cohort	Nonneutropenic adults	NA	55.7
Zhou 2017	EORTC/MSG criteria	0.7	120	Cohort	Nonneutropenic people	NA	53.3
Boch 2017	EORTC/MSG criteria	0.5	44	Cohort	Adults with MTHF	NA	52.3
Zhang 2016	EORTC/MSG criteria	0.5	94	Cohort	Adults with MTHF	NA	NA
Boch 2016	EORTC/MSG criteria	0.5	34	Cohort	Adults with MTHF	Proven/probable: 57; no IPA: 63	53.0
Fortun 2016	EORTC/MSG criteria	1	44	Cohort	Adults with ISC/COPD	NA	64.4
Lahmer 2016	EORTC/MSG criteria	0.5	49	Cohort	Adults with MTHF	59.0	57.0
Lin 2016	EORTC/MSG criteria	1	96	Cohort	Adults with MTHF	64.0	64.8
Ozger 2015	EORTC/MSG criteria	NA	44	Cohort	Nonneutropenic adults	NA	70.5
Khodavaisy 2015	EORTC/MSG criteria	1	43	Cohort	Adults with MTHF	56.5	58.8
Mohammadi 2015	EORTC/MSG criteria	0.5	70	Case control	Children with MTHF	8.4	62.5
Zhang 2015	EORTC/MSG criteria	1.19	121	Cohort	Adults with MTHF	59.3	51.2
Willinger 2014	EORTC/MSG criteria	1	47	Cohort	Patients with TR	50.6	63.6
Heng 2014	EORTC/MSG criteria	0.8	116	Cohort	Adults with HM	Proven/probable: 54; no IFD: 59	71.7
Affolter 2014	EORTC/MSG criteria	0.5	569	Cohort	Adults with IC/respiratory symptoms	54.0	66.6
Prattes 2014	EORTC/MSG criteria	1	221	Cohort	Adults with respiratory disease	NA	58.0
Hoenigl 2014	EORTC/MSG criteria	0.5	78	Case control	Adults with MTHF	58.0	67.0
Rose 2014	EORTC/MSG criteria	0.5	119	Cohort	Adults with MTHF	NA	54.5
de Mol 2013	EORTC/MSG criteria	0.5	41	Cohort	Children with MTHF	9.8	57.4
Kono 2013	NA	0.5	45	Cohort	Adults with MTHF	NA	NA
Zhang 2013	EORTC/MSG criteria	0.5	91	Cohort	Adults with COPD	64.2	80.2
Brownback 2013	EORTC/MSG criteria	0.5	143	Cohort	Adults with IC	50.4	75.0
Zhao 2013	EORTC/MSG criteria	0.5	112	Cohort	Patients with MTHF	NA	NA
Hadrich 2012	EORTC/MSG criteria	0.5	70	Case control	Patients with HM	37.6	0.7
Izumikawa 2012	Proposed enrollment criteria for prospective clinical studies of CPA by Denning were also employed, with minor modifications, in this investigation [79]	0.4	144	Cohort	Adults with MTHF	64.8	61.8
Reinwald 2012	EORTC/MSG criteria	0.5	87	Cohort	Patients with HM	NA	0.7
Tabarsi 2012	Infectious Diseases Society of America guidelines	0.5	17	Cohort	Patients with TR	34.6	NA
D'Haese 2012	EORTC/MSG criteria	0.8	251	Case control	Patients with MTHF	NA	58.2
He 2012	Based on the case definition proposed by Bulpa et al. [80]	0.8	34	Cohort	Patients with COPD	NA	NA
Bhella 2012	EORTC/MSG criteria	NA	46	Cohort	Patients with HM	NA	NA
Zhang 2011	EORTC/MSG criteria	0.5	76	Cohort	Elderly patients with lung diseases	NA	NA
Racil 2011	EORTC/MSG criteria	0.5	255	Cohort	Adults with HM	54.0	65.7
Torelli 2011	EORTC/MSG criteria	1	158	Cohort	Patients with MTHF	NA	NA
Acosta 2011	EORTC/MSG criteria	0.5	52	Cohort	Adults with MTHF	57.5	60.0
Luong 2011	EORTC/MSG criteria	0.5	150	Cohort	Patients with TR	58.4	51.3
Bergeron 2010	EORTC/MSG criteria	0.5	101	Cohort	Adults with HM	45.0	62.4
Hsu 2010	EORTC/MSG criteria	1.1	62	Case control	Patients with hematology	NA	72.6
Pasqualotto 2010	EORTC/MSG criteria	1.5	60	Cohort	Patients with TR	55.0	51.7
Park 2010	EORTC/MSG criteria	0.5	359	Cohort	Adults with MTHF	57.8	62.1
Luong 2010	EORTC/MSG criteria	3	145	Cohort	Adults with MTHF	55.0	65.0
Sarrafzadeh 2010	EORTC/MSG criteria	1.5	49	Cohort	Adults with MTHF	NA	63.3^∗^
Desai 2009	EORTC/MSG criteria	0.98	85	Cohort	Children with HM/IC	10.3	45.0
Fréalle 2009	EORTC/MSG criteria	1	64	Cohort	Adults with HM	49.2	71.9
Kimura 2009	EORTC/MSG criteria	0.5–1.3	26	Cohort	Adults with HM	70.0	80.4
Maertens 2009	EORTC/MSG criteria	1	99	Cohort	Adults with HM	53.6	NA
Shahid 2008	EORTC/MSG criteria	NA	59	Cohort	Adults with BC	58.0	91.3
Meersseman 2008	EORTC/MSG criteria	0.5	110	Cohort	Adults with MTHF	60.0	67.3
Clancy 2007	EORTC/MSG criteria	2.1	81	Cohort	Patients with TR	54.0	74.1
Husain 2007	EORTC/MSG criteria	0.5	117	Cohort	Adults with TR	52.3	44.0
Musher 2004	EORTC/MSG criteria	1	99	Cohort	Patients with allogeneic HSCT	Cases: 45.2; controls: 41.2	NA
Becker 2003	EORTC/MSG criteria	1	27	Cohort	Hematology patients	NA	NA
Danpornprasert 2010	EORTC/MSG criteria	0.5	30	Cohort	Patients with MTHF	41.0	56.7

EORTC/MSG = European Organization for Research and Treatment of Cancer/Mycoses Study Group; BALF-GM = BALF-galactomannan; IA = invasive aspergillosis; MTHF = multiple host factors; HM = hematologic malignancy; IC = immunocompromised; TR = transplant recipients; ISC = immunosuppressive conditions; COPD = chronic obstructive pulmonary disease; BC = bronchogenic carcinoma; ^∗^mean value in proven+probable+possible patients.

**Table 2:** Pooled sensitivity and specificity of the included studies for proven or probable vs. no IA.

Study	Pooled SEN (95% CI)	Pooled SPE (95% CI)
Cutoff of 0.5-1.0	0.80 (0.75-0.84)	0.88 (0.87-0.90)
Cutoff of greater than 1.0	0.84 (0.79-0.89)	0.88 (0.85-0.90)

SEN = sensitivity; SPE = specificity.

## Data Availability

There are no available data.

## Supplementary Materials

Supplementary Materials Figure S1: flow diagram of inclusion and exclusion studies. Figure S2: subgroup analysis for proven+probable vs. no (sensitivity). Figure S3: subgroup analysis for proven+probable vs. no (specificity). Figure S4: funnel plot that can reveal the publication bias of these four groups.
