# Supplementary figures and images for: Diagnostic Accuracy of Bronchoalveolar Lavage Fluid Galactomannan for Invasive Aspergillosis
Source: Biomed Res Int. 2020 Nov 30;2020:5434589. doi: 10.1155/2020/5434589 (PMC7723495; doi:10.1155/2020/5434589)

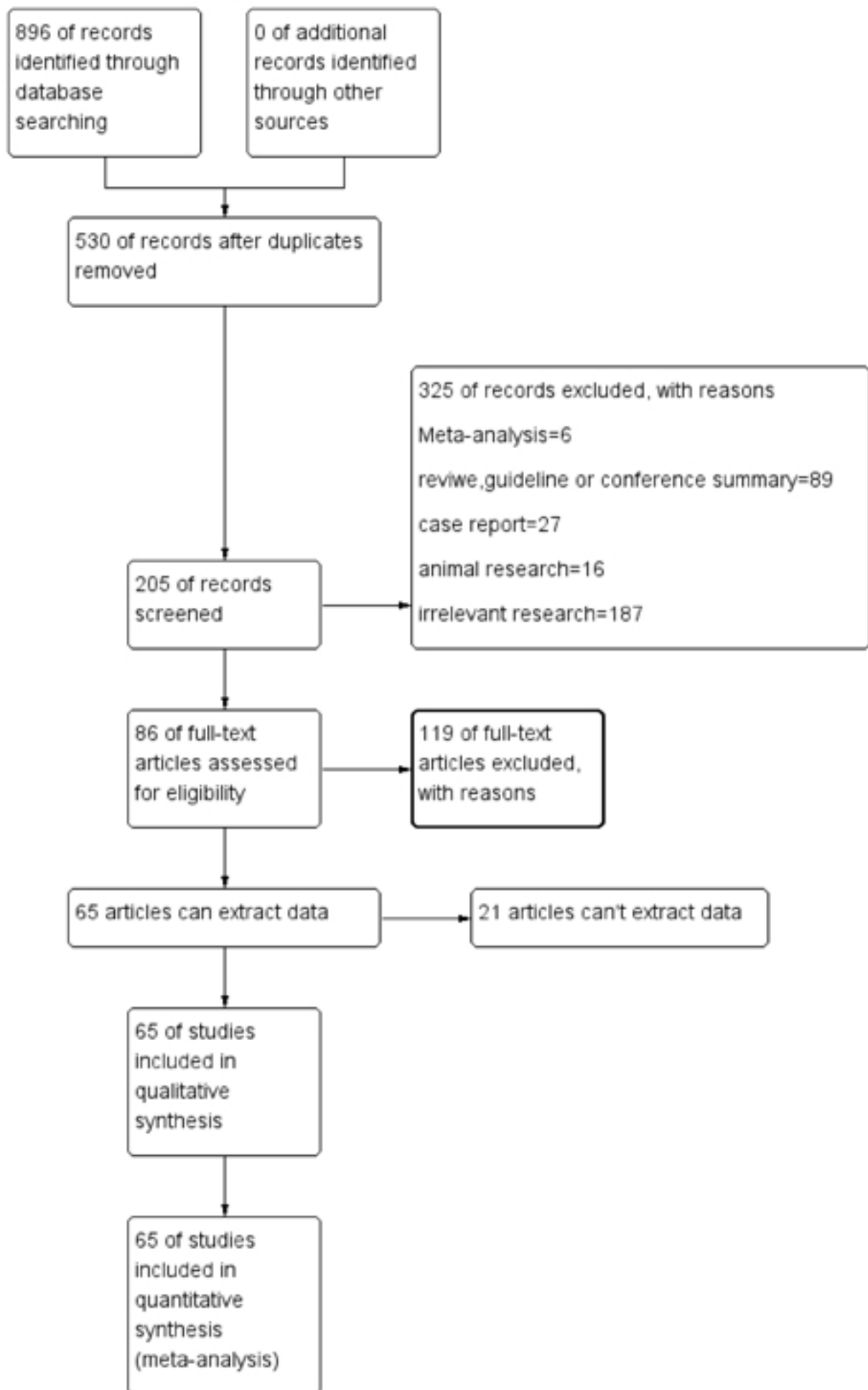

Figure S1. flow diagram of Inclusion and exclusion studies.

Supplement: Supplementary Materials — Figure S1: flow diagram of inclusion and exclusion studies. Figure S2: subgroup analysis for proven+probable vs. no (sensitivity). Figure S3: subgroup analysis for proven+probable vs. no (specificity). Figure S4: funnel plot that can reveal the publication bias of these four groups. [file 5434589.f1.pdf]
